# Mucormycosis in an Immunocompetent Patient Recovering From Dengue Fever

**DOI:** 10.7759/cureus.65212

**Published:** 2024-07-23

**Authors:** Sushmitha D. J., Kalyan Kumar Reddy Annapureddy, Nishan Poojary, Santhosh Balapanga, Bindu Kumari

**Affiliations:** 1 Otolaryngology, ClearMedi Radiant Hospital, Mysuru, IND; 2 Internal Medicine, Sri Venkateswara Medical College, Tirupati, IND; 3 General Surgery, Sri Venkateswara Medical College, Tirupati, IND

**Keywords:** mucormycosis, chronic rhinosinusitis, amphotericin b and posaconazole, case report, deviated nasal septum, immunocompetent, post dengue

## Abstract

Mucormycosis is a rare yet aggressive fungal infection. Despite its rarity, India has experienced a surge in cases during the post-COVID-19 era. The high mortality rate associated with this infection necessitates early diagnosis, intervention, and aggressive treatment. Typically, it is observed in immunocompromised patients, where the disease progresses rapidly and leads to unfavorable outcomes. However, occurrences in previously healthy individuals are not uncommon. Dengue has been occasionally associated with mucormycosis in the post-recovery phase. This case report highlights the importance of heightened clinical suspicion and early intervention in patients with recent dengue infections and chronic sinus conditions. It explores potential risk factors, such as dengue-related immune alterations, environmental exposures, and anatomical alterations that may contribute to the development of mucormycosis in otherwise healthy individuals.

## Introduction

Mucormycosis was first discovered by a German pathologist named Paltauf in 1885, and the term "mucormycosis" was coined by R. D. Baker. Mucormycosis is a very rare invasive fungal infection caused by fungi of the order Mucorales and class Zygomycetes [1]. It is transmitted through the inhalation and ingestion of spores. The spores are vasotropic, hence becoming angio-invasive and spreading locally and disseminating to various parts of the body. It can affect different parts of the body; the rhino-orbital-cerebral type is the most common and disseminated type and has a poor prognosis [2]. Fever, facial pain, facial swelling, headache, nasal discharge, nasal ulceration, and palatal ulceration are common presenting features. Manifestations of orbital spread include periorbital swelling, ophthalmoplegia, proptosis, chemosis, and reduced vision with blindness as a major complication [3]. The survival rate for rhino-orbital-cerebral disease in patients without any systemic disease is approximately 75%, with other diseases being approximately 20%, and the mortality rate can soon reach 100% if not diagnosed and treated in a timely manner [4]. Although spores are ubiquitous and present in the nasal mucosa of people, only people with poor immune systems and diabetes are at risk of developing mucormycosis [5].

It remains in the sinuses, lungs, and gut of healthy humans, without causing any disease. It usually affects individuals with a poor immune system and poor diabetic control. Other etiologies include chronic sinusitis, broad-spectrum antibiotics, stem cell therapy, skin trauma, malnutrition, ketoacidosis, neutropenia, HIV infection, organ transplantation, and previous steroid treatment [6].

Dengue is an arboviral infection caused by the dengue virus serotypes: DENVs 1-4. It spreads from one person to another via mosquito bites. Dengue virus infection results in varying degrees of pathological conditions, ranging from mild asymptomatic dengue fever (DF) to severe dengue hemorrhagic fever (DHF) and dengue shock syndrome (DSS), which may be fatal. DF is usually self-limiting and lasts five to seven days [7]. Owing to the associated comorbidities and immunocompromised status, these patients are prone to developing severe opportunistic infections. Mucormycosis is a rare opportunistic infection, but in the post-COVID-19 era, its occurrence after dengue infection is concerning and needs more research on this topic [8,9].

This case report is an attempt to understand the rare occurrence of mucormycosis in an immunocompetent patient with good glycemic control, a known case of deviated nasal septum suffering from chronic sinusitis who had recently recovered from dengue virus infection (10-12 days prior).

## Case presentation

A 42-year-old man visited a private hospital with secondary care facilities in his hometown on December 9, 2021, with a two-day history of fever. He tested positive for dengue NS1 antigen. He was later admitted for five days in the hospital where he was treated with intravenous (IV) paracetamol, IV fluids such as normal saline, and medications such as pantoprazole. There was no history of steroid use. Patients are not known to have any comorbid conditions, such as diabetes, asthma, hypertension, or any immunocompromised state in the past. He had no history of recent COVID-19 infection in the past one year. The patient did not smoke or drink alcohol. He worked as a traffic police officer. The patient had chronic sinusitis with a deviated nasal septum.

On December 15, 2021, he noticed two black spots just below the right lower eyelid near the inner canthus and mild swelling on the right cheek and right lower eyelid. He was prescribed analgesics and antiallergic medication in his hometown for the same.

As his symptoms did not improve over the course of time, he presented to our hospital which is a tertiary care hospital on December 24, 2021, with swelling around his right lower eyelid and cheek that had been increasing for the last 10 days. He also complained of mild pain and a tingling sensation on his right cheek. During the examination, two black necrotic patches were found on the skin below the right lower eyelid, along with swelling of the right lower eyelid and cheekbone. On ophthalmic examination, there was no evidence of proptosis, ophthalmoplegia, visual impartment, conjunctival chemosis, nystagmus, or fixed pupils. A CT scan of the paranasal sinuses was performed after the examination.

**Figure 1:**
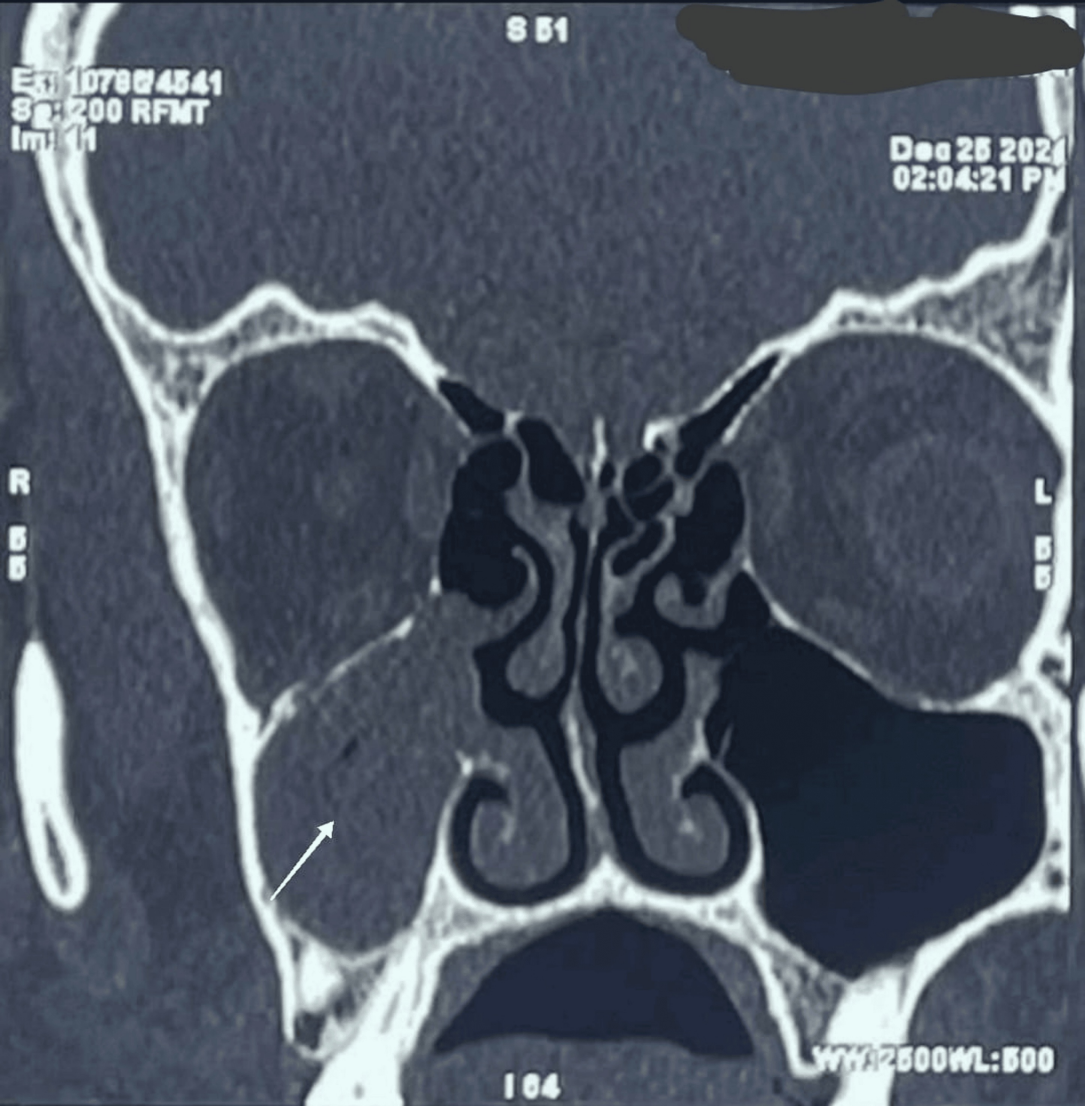
The image shows a coronal CT scan of the paranasal sinuses without contrast, revealing an isodense mass lesion in the right maxillary region. There is patchy bony destruction with scattered intraosseous air foci present in the superior wall of the right maxillary sinus.

On December 25, 2021, CT scan results revealed a deviated nasal septum towards the right, mucosal edema in the bilateral maxillary and ethmoidal sinuses, and submucosal edema in the right lower eyelid and cheek (Figure 1). The patient was later referred to the ENT department of the hospital for further management.

**Figure 2:**
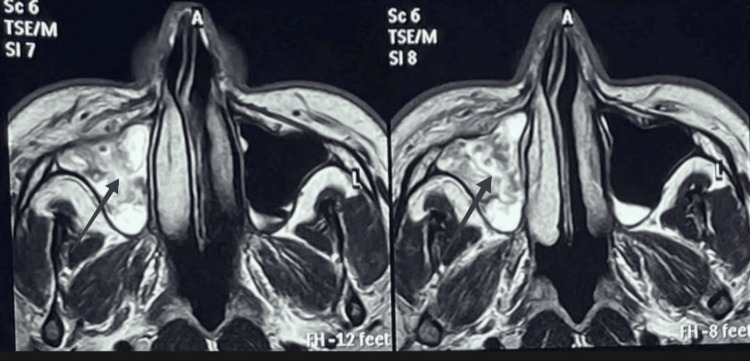
Axial T2-weighted MRI image without contrast showing hyperintense mucosal thickening and intrasinus hypointense contents in the right maxillary sinus.

As CT scan reports were inconclusive to start the treatment, an MRI was ordered, which showed mucosal thickening of the right maxillary sinus with T2 hypointense contents, thickening sclerosis, and rarefaction of the bony walls suggestive of fungal sinusitis. In addition to this, signs of orbital extension of infective sinus disease were observed, including right orbital fat stranding and soft tissue swelling (Figure 2).

**Figure 3:**
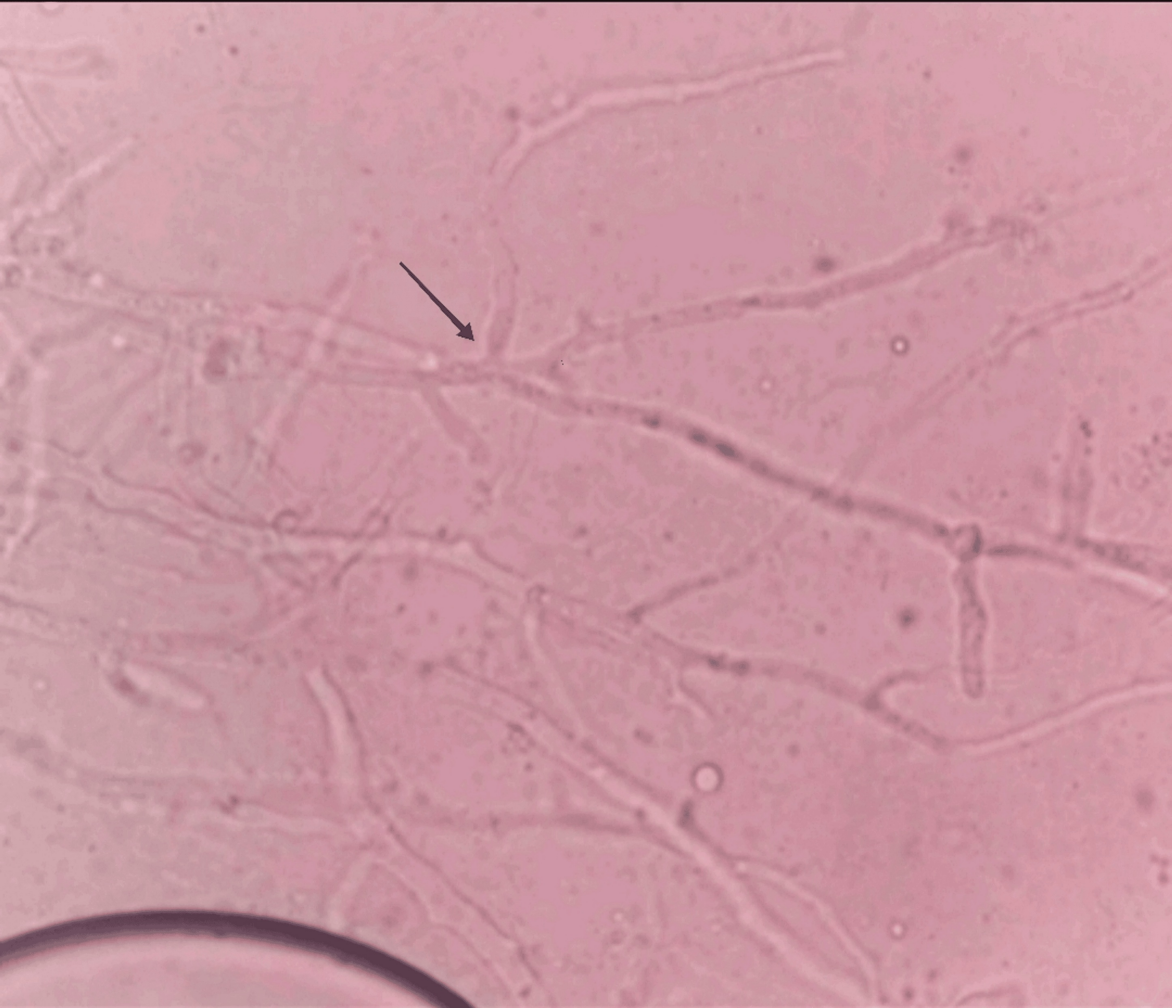
The image shows hyaline, broad, ribbon-like aseptate hyphae with wide-angle branching.

Based on the clinical suspicion and MRI reports, invasive fungal sinusitis was suspected, and functional endoscopic sinus surgery was planned. On January 7, 2022, the patient underwent a right middle meatal antrostomy with septoplasty and endoscopic debridement. During surgery, slough and necrotic tissue were observed on the right anterolateral wall, and soft tissue from the maxillary sinus, consistent with the MRI findings. The samples were then sent for histopathological examination. Periodic acid-Schiff (PAS) staining and potassium hydroxide (KOH) mounting were used to study the samples. On gross examination, the soft tissue from the maxillary sinus had multiple gray-white to gray-brown soft tissue bits, while necrotic tissue from the anterior lateral wall of the maxilla had multiple gray-white soft tissue bits. Sections from both specimens show inflammatory granulation tissue, areas of necrosis, and debris with fungal elements, which are composed of broad aseptate hyphae branching at obtuse angles (Figure 3). This was morphologically compatible with mucormycosis; therefore, the diagnosis was confirmed.

Once the diagnosis was confirmed, medical treatment was initiated for mucormycosis. Injection of liposomal amphotericin-B 5 mg/kg/day intravenously for 10 days was planned and started, but severe adverse reactions were noted, including chills, blurring of vision, and a syncopal attack following injection. Ongoing infusion was stopped, and an alternative treatment was planned. This led to a switch to IV posaconazole 300 mg BD loading dose on the first day, followed by posaconazole 300 mg OD for seven days under physician guidance, followed by oral posaconazole for 12 weeks.

**Figure 4:**
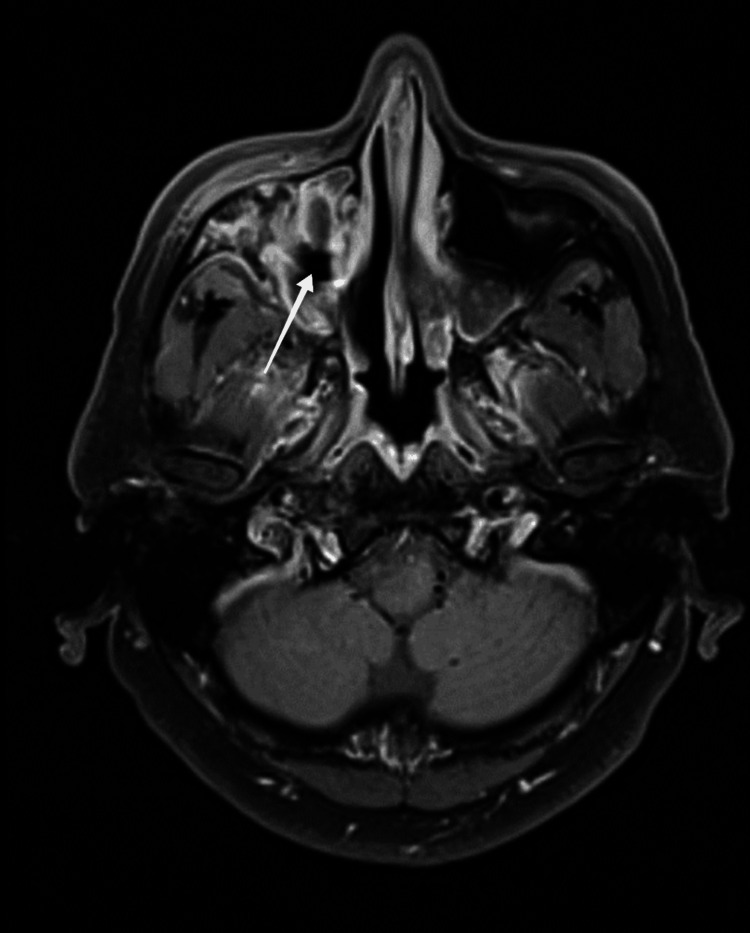
Axial T1-weighted post-contrast image showing a heterogeneous enhancement pattern in the right maxillary sinus and premaxillary region.

**Figure 5:**
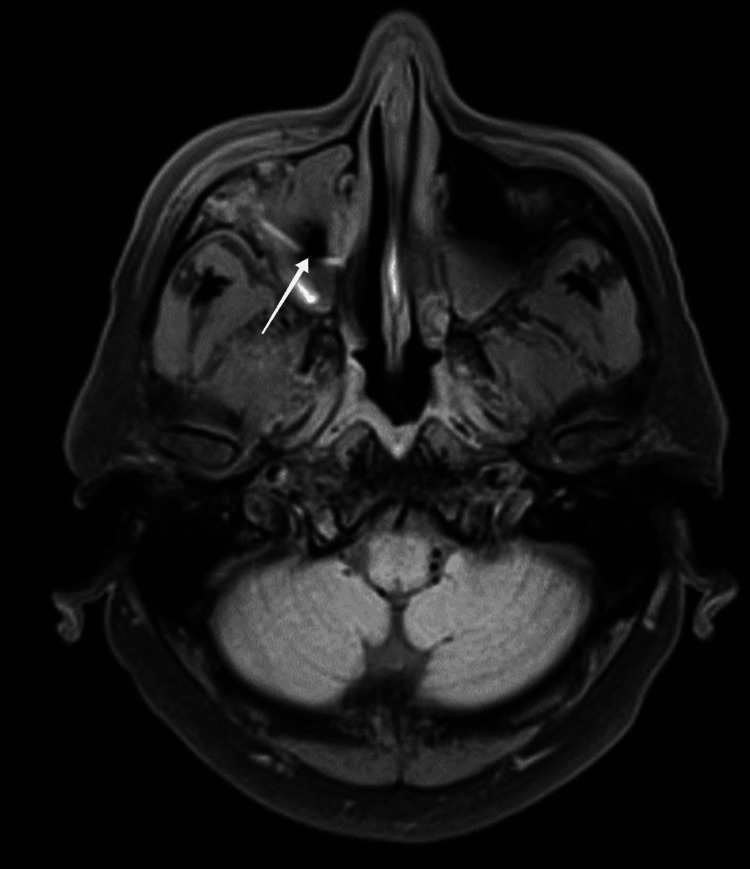
Axial T1-weighted pre-contrast image

On follow-up, the MRI was repeated on January 20, 2022, which showed circumferential mucosal thickening in the right maxillary sinus with enhancing and non-enhancing components, and mild thickening and enhancement in the superficial soft tissues in the right anterior maxillary region. MRI findings were suggestive of ongoing invasive fungal sinusitis (Figures 4-5).

On the basis of these findings, the second surgery was planned on February 1, 2023. The patient underwent endoscopic surgery with excision of the lateral and medial maxillary walls using a modified Denker’s methodology under general anesthesia, and histopathological examination revealed inflammatory nasal polyps in the tissue of the anterior and posterior walls of the maxilla. The sections studied showed polypoid fragments with a respiratory lining and subepithelial collections of inflammatory cells, composed of lymphocytes, plasma cells, a few eosinophils, and neutrophils. The histopathological examination revealed the absence of fungal elements. There was no evidence of fungal elements or neoplastic pathology. The patient’s condition improved drastically with supportive care and was later discharged on February 5, 2023, with oral posaconazole for six months. After a year, on a follow-up visit to the hospital, the patient was doing well and was disease-free.

## Discussion

Mucormycosis, colloquially known as "black fungus," is one of the most devastating infections owing to its high mortality and morbidity [10]. India had the highest number of mucormycosis cases and experienced a significant surge in the post-COVID-19 era, with approximately 40,000 cases reported by late June 2021 [11]. This alarming rise prompted several states to declare an epidemic. Despite its high incidence, there is a significant gap in the literature, with only 101 cases documented worldwide. This disparity highlights the urgent need for more research to investigate new cases and to better understand how mucormycosis infections develop and interact with other infections.

Mucormycosis typically affects immunocompromised individuals; however, it is noteworthy that 43% of cases in India involve immunocompetent patients [12,13]. Our case study highlights one such case in which a middle-aged immunocompetent patient was affected by this deadly disease. Through this study, we aimed to identify the potential risk factors that trigger infection and pathogenesis, which will help to determine the most likely etiology. This will help us to learn more about mucormycosis in immunocompetent individuals and could improve future treatment by making an earlier diagnosis.

Mucormycosis is associated with a mortality rate of ~30% [14]. The timeline plays a crucial role in treatment, as mortality can soon reach 100% if not diagnosed and treated early. However, a major challenge that hinders diagnosis is that the symptoms of mucormycosis are often nonspecific and can mimic other conditions, such as sinusitis [15]. Similarly, in our case study, the patient initially presented with vague symptoms such as swelling below the eyelid and on the cheek. A similar case was reported in Jabalpur on October 1, 2021, involving a previously healthy 20-year-old male. His symptoms began as chronic sinusitis with a headache and were initially treated with bacterial sinusitis. Following dengue infection, he developed extensive mucormycosis a week later [8].

This shows that regardless of the patient's age or immunocompromised status when symptoms such as chronic sinusitis, headache, nasal blockage, cheek swelling, and puffiness around the eyes are present; a high degree of suspicion is key for early intervention. Recognizing these early signs and promptly initiating appropriate treatment are essential to improve the outcomes of patients with mucormycosis. This highlights the fact that understanding co-infections and precipitating factors make it easy for clinicians to diagnose them at the earliest and prevent disease progression.

Co-infections significantly increase the risk of opportunistic infections such as mucormycosis. Viral and bacterial infections can impair phagocytic function, rendering the host vulnerable to opportunistic infections [16]. In particular, mucormycosis can occur during the recovery period in immunocompetent patients. To date, multiple cases of post-dengue mucormycosis have been documented [9,17].

One such case was reported on October 7, 2021, in Jabalpur, India, involving a 42-year-old female with type 2 diabetes mellitus and asthma who developed symptoms of mucormycosis six days after dengue infection [9]. Another case was reported on October 29, 2021, in Indore, India, in a 50-year-old male who developed mucormycosis while recovering from DF. Our case study highlights a similar scenario in which a middle-aged immunocompetent patient developed mucormycosis approximately one week after DF.

These cases show the importance of careful and early intervention in patients recovering from dengue, especially in those recovering from DF, to prevent serious infections, such as mucormycosis. The commonality among the cases emphasizes the importance of recognizing the heightened risk period post-dengue infection.

Several studies done to date have shown that dengue can lead to mucormycosis. Some research suggests that a "cytokine storm" triggered by the dengue virus causes lymphopenia and cytokine-induced epithelial damage, which in turn facilitates angioinvasion and dissemination of fungal elements [18]. Additionally, other studies have found that hyperferritinemia and elevated plasma levels of adhesion molecules such as soluble intercellular adhesion molecule-1 (sICAM-1), thrombospondin-1, and vinculin promote invasive fungal infections [19]. This highlights the complex interplay between the viral and fungal pathogens in immunocompetent individuals. This explains why dengue infection was the most reliable explanation as the trigger of mucormycosis in our case study.

Mignogna et al. hypothesized that chronic local insults may predispose seemingly healthy or immunocompetent individuals to develop mucormycosis [20]. In such individuals, alterations in the drainage of the paranasal sinuses, such as those caused by septal deviations, and anatomical modifications, such as concha bullosa, can facilitate the growth of these pathogens. Furthermore, studies have indicated that environmental factors, including air conditioning and hot and humid weather, contribute significantly to this condition [21]. A similar case study highlighted chronic rhinosinusitis as the most significant etiology contributing to the development of mucormycosis in a 57-year-old immunocompetent patient who presented with sinusitis symptoms without any other predisposing factors [22].

Our case correlates with this hypothesis as the patient had chronic rhinosinusitis with a deviated nasal septum. Additionally, as a traffic police officer, his job demands that he spend long hours under hot and humid conditions. He is constantly exposed to dust particles that can irritate the nasal mucosa in the long term. These situations match well with the suggested reasons, and strongly support chronic sinusitis as one of the possible etiologies of mucormycosis in this case.

The treatment plan for mucormycosis includes both surgical and medical intervention. Amphotericin B is the first-line treatment for mucormycosis followed by IV posaconazole for a sustained period [23]. In the present case, the patient developed a severe allergic reaction to amphotericin B and was switched to IV posaconazole. As amphotericin B is known for its side effects, more research is needed to find alternatives to amphotericin B and to determine the efficacy of posaconazole as a first-line drug for the treatment of mucormycosis.

## Conclusions

In our rapidly changing world in the field of infectious diseases, we have seen a lot of new infections being introduced in the last decade. Although mucormycosis is not a new infection, it has shown a recent shift, indicating its potential to become dreadful in the future. Therefore, it is crucial to understand this infection. This case is particularly important, as it highlights the development of mucormycosis in immunocompetent patients, which is not a normal occurrence. This case study highlights the diagnostic challenges of fungal sinusitis and highlights the importance of timely diagnosis and intervention in managing complications such as orbital extension. In our case, the patient had recently recovered from DF and is a known case of chronic rhinosinusitis. Recognizing symptoms early and starting both surgical and medical treatment at the right time involving the multidisciplinary team prevented any unlikely complications, such as orbital extension, in our case study.
